# Adult Status Epilepticus: A Review of the Prehospital and Emergency Department Management

**DOI:** 10.3390/jcm5090074

**Published:** 2016-08-23

**Authors:** Michael Billington, Osama R. Kandalaft, Imoigele P. Aisiku

**Affiliations:** 1 Massachusetts General Hospital, 55 Fruit St, Boston, MA 02114, USA; mbillington@partners.org; 2 Brigham and Women’s Hospital, 75 Francis St, Boston, MA 02115, USA; okandalaft@partners.org

**Keywords:** epilepsy, Status Epilepticus (SE), generalized convulsive status epilepticus (GCSE), Anti-Epileptic Drugs (AEDs), benzodiazepines

## Abstract

Seizures are a common presentation in the prehospital and emergency department setting and status epilepticus represents an emergency neurologic condition. The classification and various types of seizures are numerous. The objectives of this narrative literature review focuses on adult patients with a presentation of status epilepticus in the prehospital and emergency department setting. In summary, benzodiazepines remain the primary first line therapeutic agent in the management of status epilepticus, however, there are new agents that may be appropriate for the management of status epilepticus as second- and third-line pharmacological agents.

## 1. Introduction

Epilepsy is a common presentation globally. Historically, epilepsy has been a well-recognized clinical entity. Status epilepticus, in particular generalized convulsive status epilepticus (GCSE), while more readily clinically identifiable, has been more challenging to define. Status epilepticus has been defined in many ways: by its phenotypic presentation (generalized convulsive status epilepticus vs. many other semiologies), its etiology, its unique electroencephalographic signatures, age of initial onset, and duration. Despite some achievements in the management and treatment of epilepsy, status epilepticus (SE), in particular generalized convulsive status epilepticus (GCSE) (Table 1), and refractory SE (RSE) are associated with high morbidity and mortality. The definition of SE has evolved over the years between clinical relevance and research consistency. The evolution of the definition or nomenclature for SE illustrates the challenges of describing SE and occasionally creates confusion in the clinical environment, particularly among healthcare practitioners. The details of the definition of SE are beyond the scope of this review, however, key definitions and the most recent definition are described in Table 1.

Of the many varying presentations of seizures, GCSE is a relatively common and complex neurological emergency that confronts providers in prehospital, emergency, and critical care settings. For the purposes of this paper, we will limit ourselves to general convulsive status epilepticus (GCSE), as this is the most common presentation of status epilepticus for prehospital and emergency providers, and is the most recognizable with very limited specialized training required to identify. This narrative literature review is intended to establish the background of GCSE as a clinical entity, the epidemiology and morbidity of the condition, and the prehospital and emergency department (ED) management approaches. While the new International League against Epilepsy (ILAE) definition is laudable and we support the definition, for many reasons it is difficult to apply in the prehospital setting. Therefore, we will use the criteria of seizure lasting greater than 5 min for our definition, as this has been found to have significant clinical relevance and prognostic value.

Most of the investigations on epidemiological and management studies from the 1970s through the 1990s used a definition of a seizure lasting greater than 30 min in duration, or a period of frequent seizures without return to baseline over 30 min or longer [1]. In large part, this was based on observations of direct, irreversible neuronal injury due to excitotoxicity [2]. The lack of clinical practicality for this definition leads to the much more operational definition of SE: any seizure lasting 5 min or greater, or two or more discrete seizures [3]. Most recently, the International League against Epilepsy published a report integrating both definitions; an action that maybe academically and clinically useful, especially when considering the spectrum of care across both emergency medicine and critical care. SE is defined as any seizure or incomplete return to baseline at 5 min, which can have long term consequences, including neuronal injury, alteration of networks, or neuronal cell death beyond 30 min [2]. This rephrasing ensures both early and aggressive recognition of SE in patients in emergency and critical care settings, as well as adequate appreciation for the pathophysiological underpinnings of poor neurologic outcomes (Table 1).

## 2. Methods

A literature review from January 1947 to September 2015 of the following databases was conducted: Pubmed, OVID, MEDLINE, Up to Date, Dynamed, and MICROMEDEX. Key words in the search included “Anti-Epileptic Drugs”, “benzodiazepines”, “emergency department”, “epidemiology”, “etiology”, “Generalized Convulsive Status Epilepticus”, “guidelines”, “management”, “morbidity”, “mortality”, “non-benzodiazepine”, “pathology”, “pathophysiology”, “prehospital”, “prevalence”, “Refractory Status Epilepticus”, “seizures”, “Status Epilepticus”, “trial”. There were 21,697 papers found when searching generally for “Status Epilepticus”. Focusing on adults, prehospital setting, and emergency department we narrowed the literature reviewed to 434 articles. Abstracts from 434 were reviewed by the three authors, and 75 were selected for final inclusion.

## 3. Epidemiology

Large, but limited studies have been conducted that examine the incidence of SE, and fewer still have been performed examining long-term morbidity and mortality. The major long-term studies have reviewed SE incidence within discrete populations at specific localities, such as Richmond, VA [4], Rochester, MN [5], as well as specific European locales [6,7], but have not typically differentiated between pediatric and adult populations. Incidence has ranged widely from 3.5 to 50 episodes of GCSE per year, per 100,000 people [6,7,8], revealing a clear bimodal distribution of children less than one year of age and adults greater than 65 [9]. A recent large-scale study of multiple hospital centers has found a gradual increase in incidence across the past four decades, though this likely represents a broader definition and increased appreciation of it as a clinical entity, and possibly stronger incentives for accurate medical coding [6]. Several studies, including the original Richmond, VA study population, found a slightly increased prevalence in the African American population, and a slight male predominance [1,6].

The true morbidity and mortality of SE is also unclear. Short-term mortality has ranged from 0.5%–20% with an age >65 frequently being identified as the most significant risk factor [9,10,11]. In one recently published severity scale—the Status Epilepticus Severity Score (STESS), consisting of the variables level of consciousness, seizure type, bimodal age delineation above or below 65, and history of prior seizure [12]—the authors posited that older age, lower levels of consciousness, generalized convulsive/nonconvulsive morphologies, and absence of prior seizures were all indicative of a worse prognosis. The STESS’s prognostic predictors are based on prior literature, and their study has subsequently been prospectively validated [13]. However, the clear correlation to these findings is that the underlying etiologies of SE have much more to do with prediction of mortality, though the underlying cause is not always known in the prehospital or emergency setting. Additionally, GCSE may be a secondary manifestation of critical illness rather than a primary cause, which can make understanding the true morbidity and mortality difficult to discern from chart review [14].

While more data is needed to better understand the true epidemiology and morbidity of GCSE, what is clear is that it represents an increasingly appreciated costly clinical entity, accounting for upwards of $4 billion dollars annually in the United States [11], with seizure-related emergencies making upwards of 8% of all emergency medical services (EMS) encounters [11].

## 4. Prehospital Assessment

The initial response to an adult in status epilepticus has four critical roles: (1) to obtain any and all history available at the scene of event; (2) to secure ABCs (airway, breathing, and circulation); (3) to prevent further trauma of the seizing patient; and (4) to reverse any potentially reversible etiologies for GCSE.

Initial management begins with the bystander. We recommend and encourage the general public and those with family and friends to become familiar with the tutorial on seizure first aid. The first aid is designed for the bystander to provide care and comfort and approaches to managing the patient until first responders arrive. Initial approaches include staying with the patient while another may seek help, in order to minimize self-harm. The bystander should take note of the length of seizure and general description to the best of their ability. The bystander should attempt to stay calm and remove any objects from the area that have the potential to cause harm. These, and other measures and interventions, are further discussed on the Epilepsy Foundation website. Management continues with the dispatcher. If available, information about the patient’s prior medical history—including specifically a history of seizure, diabetes, trauma, overdose, or cardiovascular problems—should be sought from the caller. Concurrent with immediate ABCs, reports of the event from witnesses, such as recent fever, trauma, substance abuse, time of onset and duration, eye deviation, bowel/bladder incontinence, and specific motor patterns are very valuable pieces of information, but unfortunately not always available.

### 4.1. Airway

Upon arrival of emergency responders, airway, breathing, and circulation should be assessed immediately; oxygen should be applied with a goal of O_2_ saturation >92% [15]. Because of suppression of the gag reflex during SE, the patient should be placed in the left lateral decubitus position, if possible, to decrease chances of aspiration of gastric contents. Bite block application and oropharyngeal airway adjuvants may be dangerous for rescuers to apply and are not recommended; if an adjuvant airway device is needed during SE, a nasopharyngeal airway should be placed [16]. Bite block or any oropharyngeal airway adjuvants place the healthcare provider at risk of trauma (e.g., biting, tooth lacerations) during an acute seizure and potentially act as a source for upper airway obstruction.

### 4.2. Breathing

If the patient appears to be in respiratory distress, especially if cyanotic, bag-valve mask ventilation should be applied, though providers should be actively aware that the risk of aspiration increases dramatically in this setting [17]. Respiratory failure is a complication of GCSE, which may be related to medications used in treatment of status epilepticus. In the undifferentiated patient, anoxia should also be considered as a potential underlying cause [16]. Limited data exists on the clear indications for intubation in a prehospital setting by advanced cardiovascular life support (ACLS) providers, but accepted indications include respiratory depression, recurrent seizures, or depressed mental status [18]. In a large review of cases of SE, 213 of 1023 patients required intubation, though only 14 were intubated in a prehospital setting [19]. Patients who required intubation were more likely to be elderly, received higher doses of benzodiazepines, and had higher overall mortality [2]. Currently, data is lacking on clear indications for when and whom to intubate in GCSE, and discretion must be left to the prehospital providers in conjunction with individual medical control centers.

### 4.3. Circulation

Intravascular (IV) access should be attempted. Obtaining intravascular access has not been shown to significantly decrease transport time even if it slightly prolongs on-scene time [20]. It does, however, allow more choices for medication administration, including possible rapid sequence intubation (RSI) medications and administration of anti-epileptics. IV access should not delay initial treatment. Time to treatment and cessation of seizure is a key aspect of management. The RAMPART (Rapid Anticonvulsant Medication Prior to Arrival) trial demonstrates the equally efficacious use of intramuscular (IM) midazolam (Table 2) and should be incorporated into the management algorithm [21].

### 4.4. Disability/Dextrose

A point-of-care (POC) blood sugar measurement should be obtained. Hypoglycemia is a known precipitant of seizures, and if POC testing is available it should be one of the initial evaluation tests in the field. In the instance of patient with a known seizure disorder, POC testing should not delay management. If POC testing indicates blood sugar less than 80 mg/dL, dextrose (appropriate for age) should be administered [22]. While prolonged seizures and initial fall at onset can precipitate trauma, spinal precautions are no longer indicated in the seizing patient. In a review of nearly 1700 seizing patients, no spinal fractures were appreciated; patients were excluded if they were involved in an motor-vehicle collision, had fallen from >10 feet, or had sustained other obvious trauma [16].

### 4.5. Exposure

As with all prehospital scenes, prior scene safety is paramount; universal precautions should be maintained. Patient should be exposed as indicated and attention should be paid to any drug paraphernalia.

## 5. Prehospital Pharmacologic Management

Since their introduction as the mainstay of treatment of seizures in the 1960s and 1970s, benzodiazepines have remained the first line in the treatment of GCSE [23]. Importantly, despite their respiratory depressive properties, numerous studies have found the administration of per rectum (PR), Intramuscular (IM), and Intravenous (IV) benzodiazepines safe and effective when administered by prehospital providers for both adult and pediatric populations (Table 2) [2,22,24]. Pathways of administration and choice of benzodiazepine have evolved overtime. Rectal diazepam, administered both by parents and emergency responders, has been a safe and accepted practice in prehospital medicine since 1989, and is particularly well-studied in the pediatric population [25]. Doses of 0.2–0.5 mg/kg (pediatrics)/10–20 mg (adults) have been safely used, and the lipid solubility and quick absorption by the rectal mucosa produces detectable serum concentrations with 5–10 min [25]. In the prehospital setting, IV access can be challenging, particularly in the actively seizing patient. Benzodiazepines can be given in multiple alternate routes including rectally, intramuscularly, sublingually, and via intraosseous route. In adult populations, particularly in those where IV access may be difficult, rectal diazepam has been shown as safe and effective in the treatment of SE as well [25]. EMS providers should focus on the initial 0–5 min of the management of the patient.

Benzodiazepines are the primary therapeutic option in the prehospital setting. The rapidity and effectiveness for terminating seizure activity are the primary reasons for usage in any hospital setting; however, in the prehospital setting the alternative options for benzodiazepines while maintaining effectiveness makes this class of drug the mainstay for management (Table 2). One of the largest trials examining safety and efficacy of administration of intravenous benzodiazepines was the Prehospital Treatment of Status Epilepticus Trial (PHTSE Trial), comparing lorazepam, diazepam, and a placebo [28]. This randomized controlled trial looked at 258 adults in SE; patients were excluded if they were hypotensive, bradycardic, or had known history of either chronic obstructive pulmonary disease (COPD) or prolonged benzodiazepine use. Outcome measures included termination of SE by arrival to the ED as well as prehospital, in-hospital, and post-discharge complications. The authors found odds ratios of termination of SE prior to arrival of ED 5.4 (2.3–13.2) for lorazepam compared to placebo, 2.8 for diazepam (1.2–6.7), but only a trend in favor of lorazepam when compared to diazepam direct odds ratio (OR) 1.9 (0.9–4.3). Regardless of choice of benzodiazepine, no increased frequency of adverse outcomes was appreciated.

In the prehospital setting, lorazepam has limited utility as it is technically challenging to stock in ambulances, as it requires constant refrigeration [24]. Most recently midazolam—either via intramuscular, inhalation, or buccal administration—has been used successfully in the prehospital setting. The Rapid Anticonvulsant Medication Prior to Arrival Trial (RAMPART) was a double-blinded, randomized controlled trial comparing IM midazolam to IV lorazepam in both children and adults [22]. Exclusion criteria and primary/secondary outcome measures were similar to the PHTSE Trial [22]. Despite being designed as a non-inferiority trial, the authors found that IM midazolam outperformed IV lorazepam, with 73% of seizures terminating prior to arrival in the ED compared to 63% (*p* < 0.001). Additionally, no difference in adverse outcomes between the groups was appreciated. A dedicated pediatric study examining the same drug options also found IM midazolam to be equally safe and effective with slightly superior treatment times [24].

## 6. Future Directions of Prehospital Care

While prehospital EMS algorithms typically progress from immediate resuscitative methods to first-line benzodiazepine therapy, most GCSE pathways typically incorporate non-benzodiazepine class switching if seizures remain refractory to benzodiazepines. A recent clinical trial evaluated the benefits of initiating an anti-epileptic agent in the prehospital setting in conjunction with standard benzodiazepine therapy [29]. This study (SAMUKeppra) randomized patients to either clonazepam or levetiracetam versus clonazepam and a placebo, and no significant improvement was found with the addition of levetiracetam. Given limited data on the use of clonazepam in GCSE, it is difficult to draw many conclusions from this study; likely future research directions will examine different combinations of benzodiazepine/non-benzodiazepine anti-epileptic drugs (AEDs) in an attempt to maximize cessation of SE prior to arrival in the emergency department.

## 7. Emergency Department Assessment of Patients with Status Epilepticus

**Figure 1:**
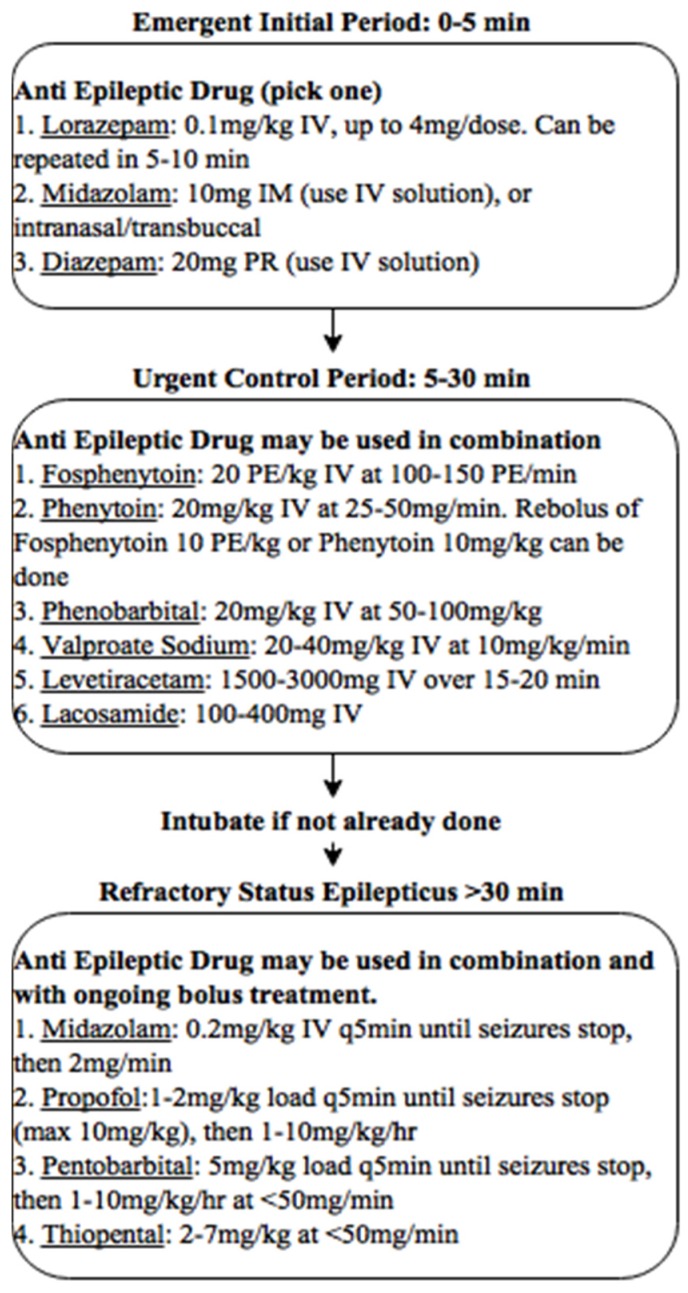
Suggested management strategy for patients.

In the ED, a reassessment of airway, breathing, and circulation must be performed and addressed as above; a particular focus must be placed on the patient given multiple doses of benzodiazepines by EMS as these patients are at greatest risk of depressive respiratory complications. A recommended management approach is described in Figure 1.

### 7.1. Consideration of Etiologies

Concurrent with evaluation, consideration to the underlying cause of SE should be taking place. Thinking through etiologies, it is helpful to subdivide categories into acute and chronic causes of SE. Acute processes can be further subdivided into (1) systemic disturbances, including electrolyte abnormalities, renal failure, sepsis, drug toxicities; and (2) central nervous system (CNS) causes including hypoxia/anoxia, infection, stroke, or head trauma [30]. Acute processes may be more difficult to control and have a higher mortality [2]; additionally, treatment requirements will be directed specifically at the underlying etiologies (e.g., intubation in refractory hypoxia, dialysis in profound uremia, antibiotics in meningitis) [31]. Chronic causes of GCSE, which may be more commonly seen, are typically in (1) patients with underlying epilepsy with breakthrough seizures provoked by nonadherence to AEDs, poor sleep hygiene, or mild provoking acute process; (2) patients withdrawing from chronic alcohol/benzodiazepine use; or (3) complications from remote CNS insults including stroke, demyelinating diseases, or autoimmune diseases [2,24,31]. These patients will typically be treated more directly with AEDs.

### 7.2. Review of Relevant Pathophysiology

The pathophysiology contributing to cessation of seizure activity in some and progression to SE and RSE is poorly understood. It is therefore useful to consider and understand the underlying etiology when deciding on treatment interventions. Since most seizures self-terminate, there is yet to be a completely understood protective mechanism, but may likely involve hyperpolarization as a mechanism for aborting most seizures. However, underpinning all seizures mechanisms, including SE, is one of two (sometimes both) processes: (1) too much excitatory glutamate neurotransmitter or (2) cessation of gamma-aminobutyric acid (GABA) inhibition [32,33]. There is evidence that the longer seizures persists, the greater the possibility of the alteration of GABA receptors that are sensitive to benzodiazepines, such that they become less effective in aborting seizures [34].

Associated physiologic parameters in SE also follow a predictable pattern [35]. In the first 30–60 min, blood pressure, serum glucose, serum lactate, cerebral blood flow, and cerebral metabolism increase. After 60 min of seizure, blood pressure, glucose, and cerebral blood flow all decrease, while the requirements of cerebral metabolism remain elevated. Ultimately, in convulsive status, a combination of respiratory acidosis and lactic metabolic acidosis may result in severe morbidity and mortality [36], but even if these processes are mitigated with medical treatment, persistent unimpeded neuronal activation can be fatal through the mechanism of excitotoxicity. On a cellular level, prolonged excitotoxicity occurs when persistent depolarization causes unimpeded intracellular neuronal influx of calcium, causing second messenger cascades leading to apoptosis or delayed cell death [34].

### 7.3. Diagnostic Testing in the ED

Recommendations of testing vary by specialist society recommendations, but there is consensus that all patients should have the following: a fingerstick glucose test; basic metabolic panel; and calcium, magnesium, and anti-epileptic medication levels if the patient is known to be on one (or multiple) [33]. Typically, in SE cases a head computed tomography (CT) scan is indicated (though brain magnetic resonance imaging (MRI) preferred, if possible, in the pediatric population) [37]. If treatment has been appropriate in the prehospital setting, plans for continuous electroencephalogram (cEEG) should be made, anticipating that some time may be required to get personnel and equipment on site.

Based on specific clinical findings (i.e., fever, known ingestion etc.), additional testing should be considered, including brain magnetic resonance imaging, lumbar puncture, liver function tests, comprehensive toxicology panels of blood and urine (looking specifically for drugs such as tricyclic antidepressants, cocaine or other sympathomimetics, alcohol, and cyclosporine), coagulation studies, blood gas, and serial troponins.

The American College of Emergency Physicians (ACEP) guidelines on the management of adults presenting to the ED with first-time seizures have suggested an algorithm produced by Dunn et al. [37] for patients presenting with their first uncomplicated generalized seizure. This algorithm, however, is limited to new onset uncomplicated seizures. There are no guidelines from ACEP on management of GCSE.

## 8. Emergency Department Medical Management

Emergency medical management should proceed in a clear stepwise progression: a first-line benzodiazepine followed by a non-benzodiazepine AED loading dose, and if still refractory, general anesthesia.

### 8.1. Benzodiazepines

Benzodiazepines presynaptically cause increased inhibition and block calcium uptake; postsynaptically they enhance GABA-nergic inhibition, and non-synaptically they increase chloride anion conductance, reducing repetitive firing of action potentials [26]. As with prehospital management, there have been numerous studies attempting to differentiate between the ideal route or drug of choice in GCSE, though unlike the prehospital setting, lorazepam is the clear first-line benzodiazepine of choice when IV access has been achieved [27], while IM/buccal midazolam remains first line when IV access has not yet been obtained [27]. Beyond efficacy, one reason lorazepam has been preferred as a first-line medication is due to its more predictable effects secondary to its smaller volume of distribution and lack of active metabolites when compared to diazepam [18]. The current recommended guidelines include administering two doses of a first-line benzodiazepine five minutes apart; if seizures continue, a non-benzodiazepine AED should be added [27].

### 8.2. Urgent Non-Benzodiazepine Anti-Epileptic Drugs (AEDs)

Non-benzodiazepine AEDs (Table 3) should be added to any patient suffering from status epilepticus, and those AEDs that have the most data researched on them include phenytoin/fosphenytoin, valproic acid [18,38], and to a lesser extent levetiracetam guidelines [38]. Ideally, they should be given within 20 min of onset of seizure. The only exception to this is if a precipitant to GCSE has been clearly identified and definitively treated [18].

#### 8.2.1. Phenytoin/Fosphenytoin

Phenytoin has been studied extensively for many years as a second-line treatment for status epilepticus and has been found effective [18]. Traditional side effects are hypotension and arrhythmias, and localized soft tissue infections, though to large extent, many of these have been more associated with additive of propylene glycol/ethanol; thus, comparatively, not as likely to present with the more water-soluble prodrug fosphenytoin [27]. For toxic seizures, these drugs may be less effective. The ACEP guidelines recommend it (with Level B evidence) as first “urgent” or second-line therapy without clear superiority to valproic acid [39]. A majority of neurocritical intensivists also chose phenytoin/fosphenytoin when surveyed for typical convulsive SE in adults [38], though it is considered slightly less preferred than valproic acid by the Neurocritical Care Society (Level IIa B evidence) [40].

#### 8.2.2. Valproic Acid

Valproic acid also has been well-established in the treatment algorithm of SE. It has been found to be efficacious and some studies have shown a trend towards superior performance in aborting SE when compared directly to fosphenytoin/phenytoin [18,41]. It has also not been associated with hypotension or other significant acute interactions during infusion. ACEP guidelines give valproic acid as initial second-line therapy (Level B evidence and Level IIa A) evidence non-benzodiazepine AED by the Neurocritical Care Society.

#### 8.2.3. Levetiracetam

Levetiracetam has become as an increasingly used agent, though much of the original literature studied is in cases of refractory seizures. More recently, some evidence has emerged that levetiracetam may be an effective alternative [42]. It also appears to have a favorable side-effect profile compared to phenytoin/fosphenytoin in that, like valproic acid, it does not have the associated risks of hypotension/dysrhythmias. Overall, compared to valproic acid and phenytoin, evidence is still emerging on comparable efficacy and larger studies are needed, ideally comparing phenytoin, valproic acid, and levetiracetam directly in the specific clinical context of post-benzodiazepine administration for the treatment of GCSE. It is considered Level C evidence by ACEP and Level IIb C by the Neurocritical Care Society.

### 8.3. Refractory Status Epilepticus

Refractory status epilepticus (RSE) is defined as persistent seizure activity, evident clinically or encephalographically, that has not responded to an initial benzodiazepine dose followed by a second-line, urgent non-benzodiazepine AED, such as phenytoin [18,43]. RSE occurs in up to 30% of all cases of SE [44] and presenting etiologies of refractory status epilepticus include causes such as acute (metabolic disturbances, sepsis, and CNS infection), drug-related (withdrawal, toxicity, and non-compliance) and chronic processes (preexisting epilepsy and CNS tumors), associated with a mortality rate of 23%–61% at discharge from hospital [45]. At this point, expert opinion is to provide general anesthesia. The current Neurocritical Care guidelines recommend midazolam (Level IIb A) over propofol (Level IIb B), while the ACEP guidelines do not differentiate between the two. In RSE, cEEG monitoring is critical to appropriate and effective management. The majority of these patients should have definitive airway management and aggressive AEDs. Urgent or emergent neurology consult and management in an intensive care unit (ICU) setting is required. A summary of recommendations according to the Neurocritical Care Society [18] is provided in Table 4.

## 9. Future Directions of Emergency and Neurocritical Care for Status Epilepticus

There is still a significant amount of work to be done regarding the optimization of management of seizures in the emergency department including understanding best practices with utilization of continuous EEG monitoring, future biomarker testing, discerning the best urgent treatment drug choice after benzodiazepines, and the best management practices of truly refractory status epilepticus.

Currently, guidelines recommend starting continuous electroencephalogram (cEEG) monitoring within one hour of onset of seizures [18] and that its use is beneficial in assessing treatment response. Continuous EEG monitoring has not been broadly utilized in the emergency department setting as a standard practice parameter, and nearly all data on cEEGs guiding management in SE have occurred in the ICU setting. The availability of cEEG monitoring and the expertise to interpret the data is highly variable. Current available data has shown discrepancies in previously accepted progression of wave forms in GCSE, calling into question how specific EEG wave forms actually are and their clinical significance [18]. Further studies are needed to best inform providers about ideal involvement of continuous EEG utilization in the emergency department.

As alluded to in the discussion of definitions above, a clear sense of when GCSE progresses from uncontrolled seizure to permanent cellular damage due excitotoxicity also needs to be better defined so as to help providers in their attempts at management as well as prognosis. While biomarkers of cerebral function have been sought in the context of SE, including neuron-specific enolase [46], and serum prolactin [47,48], a useful prognostic biomarker has yet to be identified, though would clearly be of use to the emergency and critical care provider.

Perhaps most accessible over the short term, and what is needed to help move from largely expert opinion to clear evidence, is a randomized controlled trial looking at the best performing non-benzodiazepine AED to use; or perhaps further elucidate clinical scenarios or SE subgroups that may obtain specific benefit from specific agents.

## 10. Conclusions

There has been significant advancement in SE therapy. More data is needed in the prehospital and ED assessment and management approaches. Airway management and benzodiazepines remain the cornerstone of therapy for acute cases. The primary therapeutic agent, benzodiazepines, has not changed in 50 years and there is limited data on efficacy of non–benzodiazepine AEDs, and which non-benzodiazepine AEDs should be used. Long-term longitudinal studies are needed to evaluate the impact of early therapy of GCSE and RSE on mortality and neurocognitive functional outcomes.

## Figures and Tables

**Table 1:** Evolution of definitions of status epilepticus [1,2,4].

Evolution of Definitions of Status Epilepticus	
Author and Year of Definition	Defintion
Bleck, 1991	Seizure greater than 20 min without recovery
Dodson, 1993	Seizure greater than 30 min or more than 2 seizures without recovery
Lowenstein, 1999	Seizure greater than 5 min or more than 2 seizures without recovery.
Brophy, 2012	Seizure greater than 5 min or more of continuous clinical and or EEG seizure activity or recurrent seizure activity without recovery between seizures
Trinka (ILAE Task force), 2015	Seizure that is prolonged after initiation of mechanisms to terminate the seizure within 5 min

**Table 2:** Prehospital Management of Status Epilepticus. Regardless of the choice of benzodiazepine, no increased frequency of adverse outcomes was appreciated, as per the Prehospital Treatment of Status Epilepticus (PHTSE) Trial [2,17,22,23,24,26,27].

Benzodiazepines			
Drug and Route	Clinical Application	Dose	Maximum Dose
**Lorazepam—IV/IM**	Limited use in prehospital setting due to difficulty to stock in emergency vehicles and required refrigeration.	2 mg–4 mg	8 mg
**Diazepam—IV/IM/PR**	Rectal mucosa allows rapid effectiveness within 5–10 min	5 mg–10 mg	20 mg
**Midazolam—IM/SL**	All modes of administration can be used safely in prehospital setting.	10 mg (5 mg if <50 kg)	10 mg

* PO = per os (mouth), IV = Intravenous, IM = Intramuscular, PR = per rectum, SL = sublingual.

**Table 3:** Non-benzodiazepine anti-epileptic drugs (AEDs) and their doses [17,27].

Non-Benzodiazepine Anti-Epileptic Drugs (AEDs)				
Drug and Route *	Dose **	Maximum Dose	Benefits	Disadvantages
Phenytoin—PO/IV	Oral dose of 20 mg/kg Intravenous rate at 18 mg/kg	Maximum dose of 400 mg every 2 h orally Maximum rate of 50 mg/min intravenously	Recommended by ACEP *** guidelines as first “urgent“ or second line therapy	Hypotension, arrhythmias and localized soft tissue infection
Fosphenytoin—IV/IM	Intravenous 18 PE/kg	Maximum rate of 150 PE/min	Localized soft tissue infection less likely. Fewer adverse effects vs. Phenytoin	More expensive than Phenytoin
Valproic Acid—IV	30 mg/kg	Maximum dose 60 mg/kg/day. Maximum rate of 20 mg/min	Recommended for emergent treatment of seizures and refractory Status Epilepticus	Transient local irritation at injection site
Levetiracetam—PO/IV	Oral loading dose 1500 mg Rapid intravenous loading dose is well tolerated up to doses of 60 mg/kg	Up to 3000 mg/day intravenous over 15 min	New data may show efficacy in urgent IV treatment for status and refractory status	Fatigue, dizziness, pain at infusion site

* PO = per os (mouth), IV = Intravenous, IM = Intramuscular. ** PE = Phenytoin equivalents. *** ACRP = American College of Emergency Physicians.

**Table 4:** Review of pharmacologic therapy in status epilepticus.

Review of Pharmacological Therapy in Status Epilepticus			
First Initial Line Therapy	Second Line Therapy	Third Line Therapy	Refractory Therapy
Lorezapam 0.1 mg/kg	Phenytoin 18–20 mg/kg	Propofol 1–2 mg/kg load; 10 mg/kg/h	Topiramate
Diazepam 0.15 mg/kg	Fosphenytoin 15–20 mg/kg	Phenobarbital 20 mg/kg load; 1–4 mg/kg/h	Hypothermia
	Levetiracetam 20 mg/kg	Pentobarbitaol 5–10 mg/kg load; 1–4 mg/kg/h	Ketogenic Diet
	Valproate Sodium 20–40 mg/kg	Midazolam 0.2 mg/kg load; 0.2–1 mg/kg/min	Isoflurane (Anesthesia)
		Lacosamide 200–400 mg IV bolus	Surgery
			Ketamine

## Abbreviations

The following abbreviations are used in this manuscript:

ACEP	American College of Emergency Physicians
ACLS	Advanced Cardiovascular Life Support
AED	Anti-Epileptic Drug
CNS	Central Nervous System
ED	Emergency Department
EEG	Electroencephalography(m)
cEEG	Continuous Encephalography(m)
EMS	Emergency Medical Services
GCSE	Generalized Convulsive Status Epilepticus
ICU	Intensive Care Unit
IM	Intramuscular
IV	Intravenous
OR	Odds Ratio
PHTSE	Prehospital Treatment of Status Epilepticus Trial
PO	Per Os “*By Mouth*”
RAMPART	Rapid Anticonvulsant Medication Prior to Arrival Trial
RSE	Refractory Status Epilepticus
RSI	Rapid Sequence Intubation
SE	Status Epilepticus
STESS	Status Epilepticus Severity Score

## References

[1] Delgado-Escueta A., Wasterlain C., Treiman D., Porter R. (1982). Current concepts in neurology: Management of status epilepticus. N. Engl. J. Med..

[2] Lowenstein D.H., Alldredge B.K. (1998). Status epilepticus. N. Engl. J. Med..

[3] Meldrum B., Horton R. (1973). Physiology of status epilepticus in primates. Arch. Neurol..

[4] Trinka E., Cock H., Hesdorffer D. (2015). A definition and classification of status epilepticus—Report of the ILAE Task Force on Classification of Status Epilepticus. Epilepsia.

[5] DeLorenzo R., Hauser W., Towne A. (1996). A prospective, population-based epidemiologic study of status epilepticus in Richmond, Virginia. Neurology.

[6] Hesdorffer D., Logroscino G., Cascino G., Annegers J., Hauser W. (1998). Incidence of status epilepticus in Rochester, Minnesota, 1965–1984. Neurology.

[7] Jallon P., Coeytaux A., Galobardes B., Morabia A. (1999). Incidence and case-fatality rate of status epilepticus in the Canton of Geneva. Lancet.

[8] Coeytaux A., Jallon P., Galobardes B., Morabia A. (2000). Incidence of status epilepticus in french-speaking Switzerland (EPISTAR). Neurology.

[9] Wu Y., Shek D., Garcia P., Zhao S., Johnston S. (2002). Incidence and mortality of generalized convulsive status epilepticus in California. Neurology.

[10] Dham B., Hunter K., Rincon F. (2014). The epidemiology of status epilepticus in the United States. Neurocrit. Care.

[11] Betjemann J., Josephson S., Lowenstein D., Burke J. (2015). Trends in status epilepticus-related hospitalizations and mortality: Redefined in US practice over time. JAMA Neurol..

[12] Koubeissi M., Alshekhlee A. (2007). In-hospital mortality of generalized convulsive status epilepticus: A large US sample. Neurology.

[13] Rossetti A., Logroscino G., Milligan T., Michaelides C., Ruffieux C., Bromfield E. (2008). Status epilepticus severity score (STESS): A tool to orient early treatment strategy. J. Neurol..

[14] Sutter R., Kaplan P., Ruegg S. (2013). Independent external validation of the status epilepticus severity score. Crit. Care Med..

[15] First Responder Training. http://www.epilepsy.com/get-help/services-and-support/training-programs/first-responder-training.

[16] Michael G., O’Connor R. (2011). The diagnosis and management of seizures and status epilepticus in the prehospital setting. Emerg. Med. Clin. N. Am..

[17] Stone B.J., Chantler P.J., Baskett P.J. (1998). The incidence of regurgitation during cardiopulmonary resuscitation: A comparison between the bag valve mask and laryngeal mask airway. Resuscitation.

[18] Brophy G., Bell R., Claassen J. (2012). Guidelines for the evaluation and management of status epilepticus. Neurocrit. Care.

[19] Roppolo L., Walters K. (2004). Airway management in neurological emergencies. Neurocrit. Care.

[20] Vohra T., Miller J., Nicholas K. (2015). Endotracheal intubation in patients treated for prehospital status epilepticus. Neurocrit. Care.

[21] Martin-Gill C., Hostler D., Callaway C., Prunty H., Roth R. (2009). Management of prehospital seizure patients by paramedics. Prehosp. Emerg. Care.

[22] Silbergleit R., Durkalski V., Lowenstein D. (2012). Intramuscular versus intravenous therapy for prehospital status epilepticus. N. Engl. J. Med..

[23] McArthur C.L., Rooke C.T. (1995). Are spinal precautions necessary in all seizure patients?. Am. J. Emerg. Med..

[24] Alldredge B., Gelb A., Isaacs S. (2001). A comparison of lorazepam, diazepam, and placebo for the treatment of out-of-hospital status epilepticus. N. Engl. J. Med..

[25] Dieckmann R. (1991). Rectal diazepam therapy for prehospital pediatric status epilepticus. West. J. Med..

[26] Dunn M., Breen D., Davenport R., Gray A. (2005). Early management of adults with an uncomplicated first generalised seizure. Emerg. Med. J..

[27] Treiman D., Meyers P., Walton N. (1998). A comparison of four treatments for generalized convulsive status epilepticus. N. Engl. J. Med..

[28] Fitzgerald B., Okos A., Miller J. (2003). Treatment of out-of-hospital status epilepticus with diazepam rectal gel. Seizure.

[29] Welch R., Nicholas K., Durkalski-Mauldin V. (2015). Intramuscular midazolam versus intravenous lorazepam for the prehospital treatment of status epilepticus in the pediatric population. Epilepsia.

[30] Navarro V., Dagron C., Elie C. (2016). Prehospital treatment with levetiracetam plus clonazepam or placebo plus clonazepam in status epilepticus (SAMUKeppra): A randomised, double-blind, phase 3 trial. Lancet Neurol..

[31] Towne A., Pellock J., Ko D., DeLorenzo R. (1994). Determinants of mortality in status epilepticus. Epilepsia.

[32] Spatola M., Novy J., Pasquier R.D., Dalmau J., Rossetti A. (2015). Status epilepticus of inflammatory etiology: A cohort study. Neurology.

[33] Huff J., Fountain N. (2011). Pathophysiology and definitions of seizures and status epilepticus. Emerg. Med. Clin. N. Am..

[34] Scott R., Surtees R., Neville B. (1998). Status epilepticus: Pathophysiology, epidemiology, and outcomes. Arch. Dis. Child..

[35] Kapur J., Macdonald R. (1997). Rapid seizure-induced reduction of benzodiazepine and Zn^2+^ sensitivity of hippocampal dentate granule cell GABAA receptors. J. Neurosci..

[36] Fountain N., Lothman E. (1995). Pathophysiology of status epilepticus. J. Clin. Neurophys..

[37] Walker M. (2005). Status epilepticus: An evidence based guide. Br. Med. J..

[38] Huff J., Melnick E., Tomaszewski C. (2014). Clinical policy: Critical issues in the evaluation and management of adult patients presenting to the emergency department with seizures. Ann. Emerg. Med..

[39] Hung O., Shih R. (2011). Antiepileptic drugs: The old and the new. Emerg. Med. Clin. N. Am..

[40] Riviello J.J., Claassen J., LaRoche S. (2013). Treatment of status epilepticus: An international survey of experts. Neurocrit. Care.

[41] Limdi N., Shimpi A., Faught E., Gomez C., Burneo J. (2005). Efficacy of rapid IV administration of valproic acid for status epilepticus. Neurology.

[42] Misra U., Kalita J., Patel R. (2006). Sodium valproate vs phenytoin in status epilepticus: A pilot study. Neurology.

[43] Berning S., Boesebeck F., van Baalen A., Kellinghaus C. (2009). Intravenous levetiracetam as treatment for status epilepticus. J. Neurol..

[44] Tripathi M., Vibha D., Choudhary N. (2010). Management of refractory status epilepticus at a tertiary care centre in a developing country. Seizure.

[45] Mayer S.A., Claassen J., Lokin J., Mendelsohn F., Dennis L.J., Fitzsimmons B.F. (2002). Refractory status epilepticus: Frequency, risk factors, and impact on outcome. Arch. Neurol..

[46] Nei M., Lee J., Shanker V., Sperling M. (1999). The EEG and prognosis in status epilepticus. Epilepsia.

[47] DeGiorgio C., Heck C., Rabinowicz A., Gott P., Smith T., Correale J. (1999). Serum neuron-specific enolase in the major subtypes of status epilepticus. Neurology.

[48] Aydin S., Dag E., Ozkan Y., Arslan O., Koc G., Bek S., Kirbas S., Kasikci T., Abasli D., Gokcil Z. (2011). Time-dependent changes in the serum levels of prolactin, nesfatin-1 and ghrelin as a marker of epileptic attacks young male patients. Peptides.

